# Ellagic Acid Reduces Cadmium Exposure-Induced Apoptosis in HT22 Cells via Inhibiting Oxidative Stress and Mitochondrial Dysfunction and Activating Nrf2/HO-1 Pathway

**DOI:** 10.3390/antiox13111296

**Published:** 2024-10-25

**Authors:** Yue Liu, Chunhong Chen, Zhihui Hao, Jianzhong Shen, Shusheng Tang, Chongshan Dai

**Affiliations:** 1 National Key Laboratory of Veterinary Public Health and Safety, College of Veterinary Medicine, China Agricultural University, Beijing 100193, China; s20203050760@cau.edu.cn (Y.L.); s20223050899@cau.edu.cn (C.C.); haozhihui@cau.edu.cn (Z.H.); sjz@cau.edu.cn (J.S.); 2 Technology Innovation Center for Food Safety Surveillance and Detection (Hainan), Sanya Institute of China Agricultural University, Sanya 572025, China; 3 Key Biology Laboratory of Chinese Veterinary Medicine, Ministry of Agriculture and Rural Affairs, Beijing 100193, China

**Keywords:** cadmium, ellagic acid, JNK pathway, oxidative stress, apoptosis, Nrf2/HO-1 pathway

## Abstract

Exposure to cadmium sulfate (CdSO_4_) can lead to neurotoxicity. Nevertheless, the precise molecular mechanisms underlying this phenomenon remain unclear, and effective treatment strategies are scarce. This study explored the protective effects of ellagic acid (EA), a natural polyphenolic compound, against CdSO_4_ exposure-induced neurotoxicity in HT22 cells and the underlying molecular mechanisms. Our findings demonstrated that exposure of HT22 cells to CdSO_4_ resulted in apoptosis, which was effectively reversed by EA in a dose-dependent manner. EA supplementation also decreased reactive oxygen species (ROS) and mitochondrial ROS production, reduced malondialdehyde (MDA) levels, and restored the activities of superoxide dismutase (SOD) and catalase (CAT). Additionally, EA supplementation at 5–20 μM significantly counteracted Cd-induced the loss of mitochondrial membrane potential and the decrease of ATP and reduced the ratio of Bax/Bcl-2 and cleaved-caspase-3 protein expression. Furthermore, EA supplementation resulted in the upregulation of Nrf2 and HO-1 protein and mRNAs while simultaneously downregulating the phosphorylation of JNK and p38 proteins. The pharmacological inhibition of c-Jun N-terminal kinase (JNK) partially attenuated the activation of the Nrf2/HO-1 pathway induced by CdSO_4_ and exacerbated its cytotoxic effects. In conclusion, our findings suggest that ethyl acetate (EA) supplementation offers protective effects against CdSO_4_-induced apoptosis in HT22 cells by inhibiting oxidative stress and activating the Nrf2 signaling pathway. Furthermore, the activation of the JNK pathway appears to play a protective role in CdSO_4_-induced apoptosis in HT22 cells.

## 1. Introduction

Cadmium (Cd) is a prevalent non-essential heavy metal known to have highly toxic effects on both humans and animals [1]. Cd has been ranked 7th in the hazardous waste priority list according to the U.S. Environmental Protection Agency’s Superfund, based on its level of toxic risk [1]. The World Health Organization and The International Agency for Research on Cancer (IARC) have both classified Cd as a human carcinogen [2]. Previous studies from animal models and in vitro cell models showed that Cd exposure could cause multiple toxic effects, including hepatoxicity, nephrotoxicity, reproductive toxicity, immunotoxicity, and gastrointestinal toxicity [3,4,5,6,7]. Cd has the potential to penetrate the blood-brain barrier (BBB), leading to its accumulation in the central nervous system and ultimately causing neurotoxicity. A recent epidemiological investigation showed that environmental exposure to Cd may be a risk factor for Alzheimer’s disease in humans [8]. Epidemiologic studies have indicated that Cd exposure is positively corrected with Alzheimer’s disease [9,10]. For example, a meta-analysis showed that Cd concentrations in whole blood, serum, or plasma samples of Alzheimer’s disease patients were significantly higher than those in controls [10]. In a mouse model, Cd exposure could result in abnormal neurobehavioral changes (such as movement disorders, attention deficits, headaches, and cognitive dysfunction) and pathological injury in brain tissues [11,12,13]. Hence, investigating the potential health risks of environmental Cd exposure in the nervous system and understanding the underlying molecular mechanisms are of great importance.

The precise molecular mechanisms underlying neurotoxicity induced by Cd exposure are not yet fully elucidated. Deng et al. found that chronic exposure to Cd could result in cognitive function impairment in male mice by promoting mitochondrial fission and dysfunction within brain tissues [1]. It was reported that Cd exposure could affect the function of mitochondria by blocking the electron transport chain and reducing membrane potential in neuronal cells in vitro [8,14]. A prior study indicated that Cd exposure at 2.5–20 μM could induce excessive production of reactive oxygen species (ROS) and decrease the abilities of the antioxidant defense system, triggering oxidative stress damage and mitochondrial dysfunction in PC12 cells and rat primary cerebral cortical neurons [15]. Moreover, it was also documented that Cd exposure could also trigger the activation of p53, sirtuin-1 (SIRT1), mitogen-activated protein kinase (MAPK), PKR-like ER kinase (PERK)/activating transcription factor 4 (ATF4), and ferroptosis pathways in neuronal cells and human peripheral blood lymphocytes [13,15,16,17,18,19].

**Figure 1:**
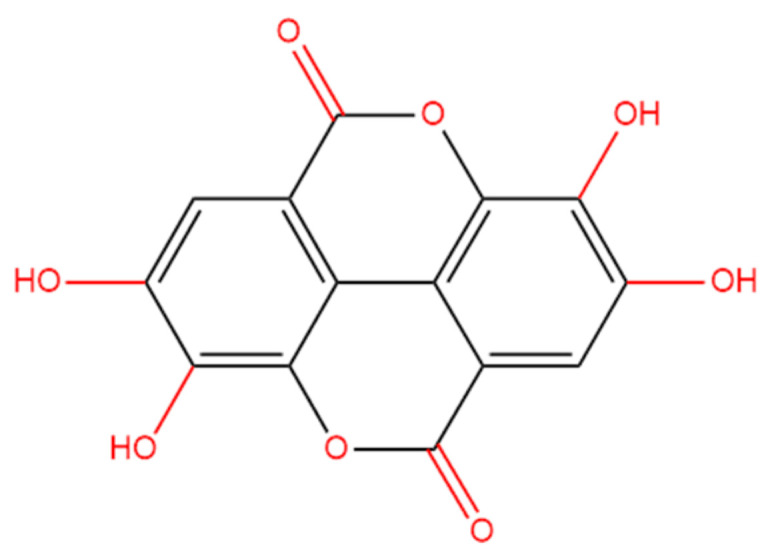
The structure of ellagic acid (2,3,7,8-tetrahydroxychromeno (5,4,3-cde) chromene-5,10-dione, C_14_H_6_O_8_).

Ellagic acid (EA) is a polyphenolic compound (2,3,7,8-tetrahydroxychromeno (5,4,3-cde) chromene-5,10-dione, C_14_H_6_O_8_; Figure 1), and it could be found in multiple gallnuts and fruits, such as pomegranates, black currants, raspberries, and mangoes [20]. The literature reports that EA exhibits various biological activities, such as anti-inflammatory, antiviral, antioxidant, antimicrobial, and immune-regulatory effects [21]. A mouse model has demonstrated that oral EA administration could effectively attenuate rotenone exposure-induced dopamine neuronal damage via targeting activation of the Nrf2 pathway [22]. Goudarzi et al. study showed that oral EA supplementation at the doses of 10 and 30 mg/kg per day for successive twenty-one days can effectively attenuate sodium arsenate-caused neurotoxicity by inhibiting oxidative stress and neuroinflammation in the brain tissue of rats [23]. The current study aims to examine the protective effects of EA supplementation against Cd exposure-induced toxicity in HT22 cells, a mouse hippocampal neuronal cell line. Additionally, we explore the underlying molecular mechanisms.

## 2. Materials and Methods

### 2.1. Chemical and Reagents

Cadmium sulfate (CdSO_4_) (purity ≥ 98%) and ellagic acid (purity more than 96%) were both purchased from Aladdin (Shanghai, China). SB203580 (a special inhibitor of p38 MAPK), SP600125 (a special inhibitor of JNK), dimethyl sulfoxide (DMSO), 1% (*v*/*v*) penicillin-streptomycin, phenylmethanesulfonylfluoride (PMSF), pan-caspase inhibitor (Z-VAD-FMK), and N-acetylcysteine (NAC) were supplied by Beyotime Biotechnology Company (Haimen, Jiangsu, China). ASN007 (a special inhibitor of ERK) was purchased from MCE (Shanghai, China). Mito-TEMPO was purchased from Santa Cruz Biotechnology (Dallas, TX, USA). All other chemical reagents used in the present study were at analysis grade.

### 2.2. Cell Viability and Treatment

The mouse HT22 and rat PC12 cell lines were both obtained from the Shanghai Cell Bank of the Chinese Academy of Sciences (Shanghai, China). Cells were cultured in Dulbecco’s Modified Eagle’s Medium (DMEM) supplemented with 10% (*v*/*v*) fetal bovine serum (FBS) and 1% (*v*/*v*) penicillin-streptomycin under controlled conditions in a cell culture incubator (Thermo Fisher Scientific, Shanghai, China) maintained at 5% CO_2_ and 37 °C.

### 2.3. Measurement of Cell Viabilities

Cell viabilities were determined using a CCK-8 detection kit (Nanjing KeyGEN Biotech Co., Ltd., Nanjing, China), according to the instructions provided by the manufacturer. Briefly, A 96-well cell culture plate was used. A total of 1 × 10^4^ cells were seeded per well. After 24 h cultivation, cells were treated with various concentrations of CdSO_4_ (0.625, 1.25, 2.5, 5, 10, 20, and 40 μM) or EA (5, 10, 20, 40, and 80 μM) for another 24 h. After the treatment, cells were incubated with 10 μL of CCK-8 reagent for 1 h. Then, cell viabilities were determined at 450 nm using a microplate reader (Tecan Trading AG, Kanton Zürich, Switzerland).

We also tested the effects of EA, antioxidant NAC, and JNK inhibitor (i.e., SP600125) supplementation on Cd exposure-induced cytotoxicity in HT22 cells. In brief, cells were pre-treated with EA at the doses of 5, 10, and 20 μM, NAC at the dose of 2.5 mM, or the JNK inhibitor at a concentration of 10 μM, as well as SB203580 and ASN007 at concentrations of 5 and 10 μM for a duration of 2 h. Subsequently, the cells were co-treated with CdSO_4_ at a concentration of 10 μM for an additional 24 h. Finally, cell viability was assessed.

### 2.4. LDH Measurement

Shown in Supplementary Material and Methods.

### 2.5. Measurement of Cell Apoptosis

Cell apoptosis analysis was performed using a commercial Annexin V-FITC/PI Apoptosis Detection Kit (Vazyme Biotech Co., Ltd., Nanjing, China) by flow cytometry analysis (Becton Dickinson, San Jose, CA, USA), according to the description in a previous study [24]. Meanwhile, the nuclear morphological alterations were assessed using the Hoechst 33,342 staining technique. The aggregation of chromosomes and the formation of apoptotic bodies were identified as indicators of cell apoptosis and were quantified accordingly.

### 2.6. Measurement of Intracellular ROS Levels and Oxidative Stress Biomarkers

Shown in Supplementary Material and Methods.

### 2.7. Measurement of Mitochondrial ROS

The levels of mitochondrial ROS were measured using the MitoSOX staining method (M36008, Invitrogen, Shanghai, China), according to our previous study [25]. In brief, cells were pre-treated with EA at the dose of 20 μM or Mito-TEMPO at 5 μM for 2 h, then co-treated with or without CdSO_4_ at 10 μM for an additional 24 h. Then, cells were washed twice with PBS and incubated with MitoSOX Red (at 5 μM) plus 4′-6-diamidino-2-phenylindole (DAPI) for an additional 30 min. Finally, cells were washed using PBS twice, and a laser confocal microscope (Leica Microsystems, Hessen, Germany) was used to observe the changes in mitochondrial ROS.

### 2.8. Measurement of Intracellular ATP Levels

Show in Supplementary Material and Methods.

### 2.9. Western Blotting

The expression of proteins was analyzed using Western Blotting, according to the detailed protocol provided in a previous study [26]. Primary rabbit polyclonal antibodies targeting phosphorylated (p)-p38 (Thr180/Tyr182), p-JNK (Thr183/Tyr185), p-ERK1/2 (Thr202/Tyr204) (1:1000 dilution; CST company, Beverly, MA, USA), Nrf2, HO-1, Bax, Bcl-2, and caspase-3 (1:1000 dilution; Proteintech, Chicago, IL, USA), and mouse monoclonal antibody against β-actin (1:1000 dilution; Santa Cruz, CA, USA) were used. A detailed protocol is shown in the Supplementary Material and Methods.

### 2.10. qRT-PCR Analysis

The RNA samples were isolated using a commercial Fast Pure^®^ Cell/Tissue Total RNA Isolation Kit V2 (Vazyme Biotech Co., Ltd.). The details are shown in Supplementary Material and Methods.

### 2.11. Statistical Analysis

All data were processed using GraphPad Prism 9.0 software. A nonlinear model-based approach from GraphPad Prism 9.0 software was employed for the calculation of IC_50_. All data were shown as means ± standard deviation (SD) unless specially stated. The differences between any two groups were performed under Tukey’s multiple comparison test of 1-way analysis of variance. A *p*-value < 0.05 was considered as considered statistically significant.

## 3. Results

### 3.1. EA Supplementation Attenuates CdSO_4_-Induced Loss of Cell Viability and the Release of LDH in HT22 Cells

**Figure 2:**
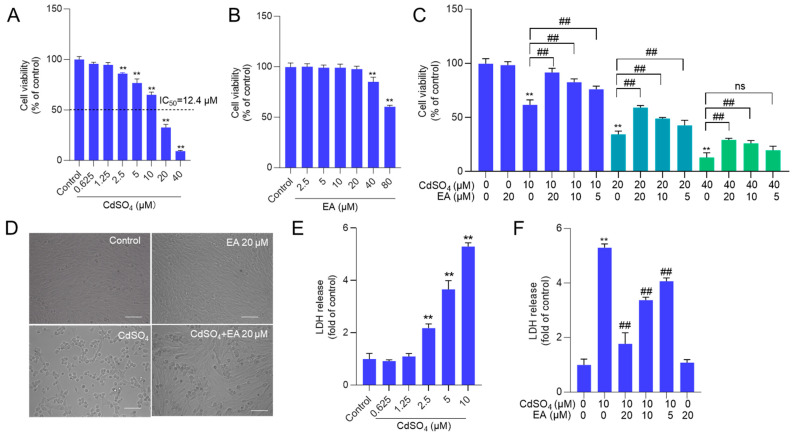
The cell viability and LDH level in HT22 cells exposed to ellagic acid (EA), CdSO_4,_ or combination. A and B cells were treated with CdSO_4_ (at 0.625, 1.25, 2.5, 5, 10, 20, and 40 μM) (**A**) or EA at various concentrations (at 2.5, 5, 10, 20, and 40 μM) (**B**) for 24 h, the cell viability was examined using the CCK-8 method. (**C**) cells were pre-treated with EA at the doses of 5, 10, or 20 μM for 2 h; then, cells were co-treated with CdSO_4_ at concentrations of 10, 20, or 40 μM for an additional 24 h. After treatment, the cell viability was examined using the CCK-8 method. (**D**) the morphology changes of HT22 cells exposed to CdSO_4_ treatment at 10 μM with or without EA at 20 μM. Bar = 50 μm. (**E**,**F**), the levels of LDH in the medium. HT22 cells were treated with the same conditions as cells in (**A**,**B**), respectively. All results were shown in mean ± SD (n = 3). Compared to the untreated control group, ** *p* < 0.01; compared to the CdSO_4_ only-treated group, ^##^ *p* < 0.01. ns, no significant.

We first established the toxic model of CdSO_4_ cytotoxicity in HT22 cells. The results showed that CdSO_4_ treatment for 24 h dose-dependently decreased cell viability in HT22 cells. As shown in Figure 2A, CdSO_4_ exposure at the concentrations of 2.5, 5, 10, 20, and 40 μM for 24 h reduced the cell viabilities to 86%, 77%, 65%, 33%, and 9% (all *p* < 0.01), respectively, and the half maximal inhibitory concentration (IC_50_) was 12.4 μM. Additionally, we also assessed the toxic effects of EA. As shown in Figure 2B, EA treatment at 5–20 μM for 24 h did not change the cell viabilities of HT22 cells, but at 40 and 80 μM, the cell viabilities were decreased to 85% and 61% (both *p* < 0.01), respectively, compared to the untreated cells. Therefore, the concentrations of EA at 5–20 μM in the following investigation were used. Then, EA treatment at 5–20 μM could effectively decrease CdSO_4-_induced cytotoxicity in HT22 cells, and it was in a dose-dependent manner. As shown in Figure 2C, EA treatment at concentrations of 5, 10, and 20 μM increased the cell viabilities to 77%, 84%, and 92% (all *p* < 0.01), respectively, compared to 10 μM CdSO_4_ group, increased the cell viabilities to 43%, 49%, and 59% (all *p* < 0.01), respectively, compared to 20 μM CdSO_4_ group; and increased cell viabilities to 20%, 26% (*p* < 0.01), and 29% (*p* < 0.01), respectively, compared to 40 μM CdSO_4_ group. Correspondingly, CdSO_4_ treatment at 10 μM for 24 h could result in marked morphology changes, such as cell shrinkage and lysis. EA co-treatment at 20 μM could improve these changes (Figure 2D). Similarly, in PC12 cells, CdSO_4_ exposure at 5 μM significantly decreased the cell viabilities to 56% (*p* < 0.01) and morphological changes, which were ameliorated by EA supplementation at the concentrations of 10 and 20 μM (Supplementary Figure S1). The corresponding cell viability increased to 66% and 74% (*p* < 0.05 or 0.01), respectively.

Additionally, a sub-IC_50_ concentration of CdSO_4_ at 10 μM was used to investigate the potential mechanisms of EA. The levels of LDH released by CdSO_4_ exposure and the protective effects of EA were assessed. Compared to the control group, CdSO_4_ exposure for 24 h could dose-dependently induce the release of LDH in the medium. The levels of LDH in the 2.5, 5, and 10 μM CdSO_4_ treatment groups were significantly increased to 2.2-, 3.7-, and 5.5-fold (all *p* < 0.01) (Figure 2E). Pre-treatment with EA at doses of 5, 10, and 20 μM significantly reduced LDH levels to 4.1-, 3.4-, and 1.7-fold (all *p* < 0.01), respectively, compared to the group treated with CdSO_4_ only (Figure 2F). Treatment with EA alone at a concentration of 20 μM for 24 h did not result in changes to the LDH levels of HT22 cells, compared to the untreated control group.

### 3.2. EA Supplementation Ameliorates CdSO_4_-Induced Cell Apoptosis in HT22 Cells

**Figure 3:**
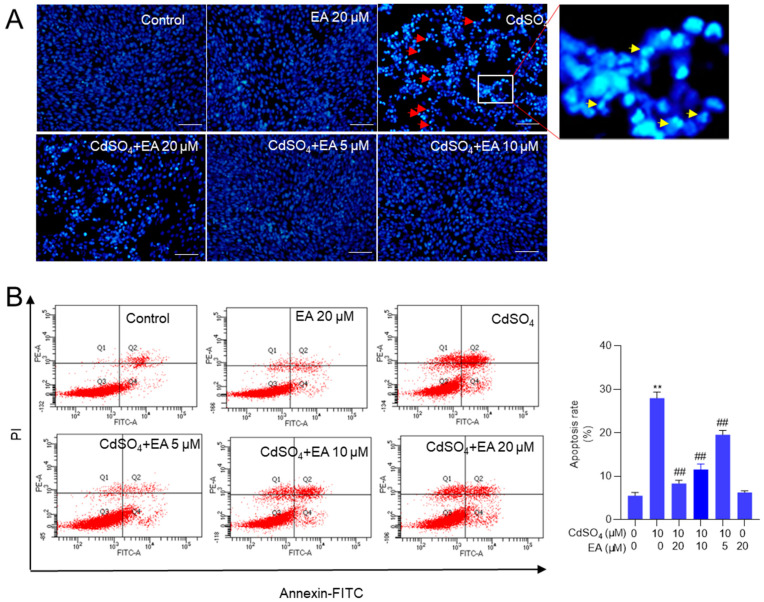
EA supplementation attenuates CdSO_4_-induced cell apoptosis in HT22 cells. Cells were pre-treated with ellagic acid (EA) at final concentrations of 5, 10, or 20 μM for 2 h, then co-treated with CdSO_4_ at 10 μM for 24 h. (**A**) nuclear morphology changes were assessed using the Hoechst 33,342 staining method. Bar = 50 μm. (**B**) apoptotic rates were quantified through Annexin V-FITC staining combined with flow cytometry analysis. Representative images of the flow cytometry analysis (left) and quantitative results (right) are displayed. All data are presented as mean ± SD (n = 3). ** *p* < 0.01 compared to the control group; ^##^ *p* < 0.01 compared to the CdSO_4_ only-treated group. The red arrow indicates chromosomal aggregation and the yellow arrow indicates nuclear fragmentation.

Exposure to CdSO4, when compared to the control group, resulted in significant changes in nuclear morphology. Figure 3A shows the presence of chromosomal aggregation and nuclear fragmentation in the 10 μM CdSO_4_ treatment group, effects that were effectively reversed by pre-treatment with EA. Similarly, EA pre-treatment at doses of 5, 10, and 20 μM markedly prevented Cd-induced cell apoptosis, leading to a decrease in apoptotic rates from 29% to 20%, 12%, and 8% (all *p* < 0.01) respectively, compared to that in the 10 μM CdSO_4_ only group (Figure 3B). Treatment with EA alone at a concentration of 20 μM for 24 h did not result in alterations in apoptotic rates in HT22 cells compared to those in the untreated control group. Consistently, our results also found that caspase pan-inhibitor Z-VAD-FMK treatment at the dose of 20 μM could markedly inhibit CdSO_4_-induced apoptosis (Supplementary Figure S2). These data suggested that apoptosis plays a critical role in CdSO_4_-induced cell death and the protective effects of EA.

Similarly, we also found that Cd exposure at 5 μM increased the apoptosis rates to 43% (*p* < 0.01) compared to the control group. EA supplementation at 5, 10, and 20 μM decreased the apoptosis rates to 34%, 23%, and 17% (all *p* < 0.01), respectively (Supplementary Figure S3). EA supplementation at 20 μM for 24 h did not affect the apoptosis rates in PC12 cells compared to the untreated control group (Supplementary Figure S3).

### 3.3. EA Supplementation Attenuates CdSO_4_-Induced ROS Production and Oxidative Stress Damage in HT22 Cells

**Figure 4:**
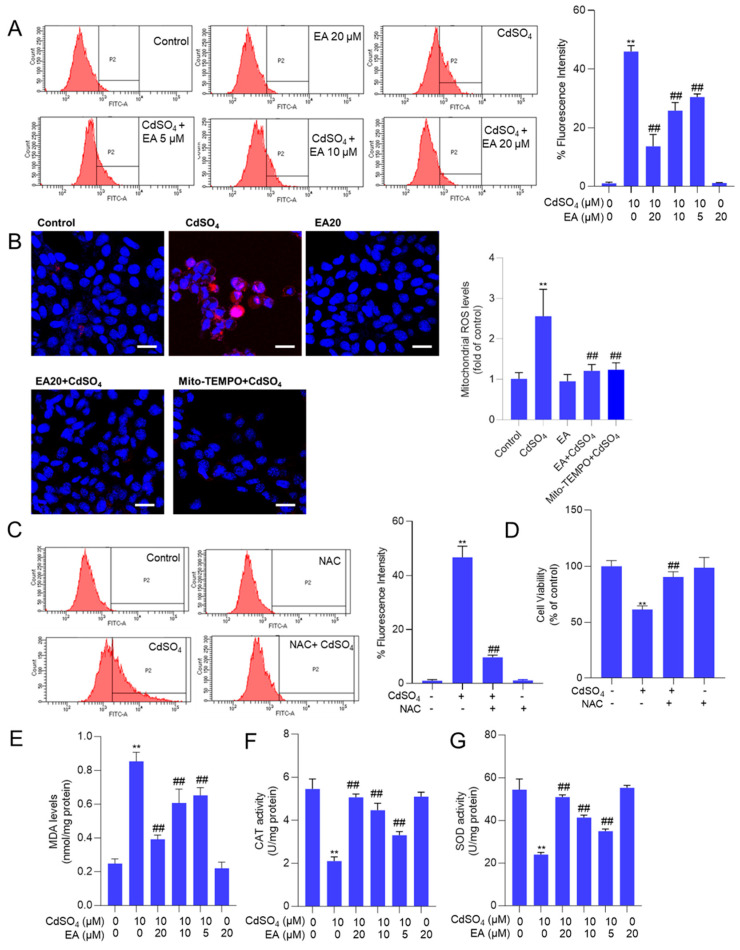
EA supplementation attenuates CdSO_4_-induced ROS production and oxidative stress damage in HT22 cells. Cells were pre-treated with ellagic acid (EA) at 5, 10, or 20 μM for 2 h, followed by co-treatment with CdSO_4_ at a final concentration of 10 μM for 24 h. (**A**) the levels of reactive oxygen species (ROS) were assessed using DCFH-DA staining in conjunction with flow cytometry analysis, with representative images displayed (on the left) and quantitative analysis (on the right). (**B**) levels of mitochondrial ROS in HT22 cells. Cells were pre-treated with EA at 20 μM or Mito-TEMPO at 5 μM for 2 h, followed by co-treatment with CdSO_4_ at 10 μM for an additional 24 h. Representative images (left) and quantitative results (right) are displayed. Bar = 25 μm. (**C**,**D**) antioxidant N-acetylcysteine (NAC) supplementation attenuates CdSO_4_ exposure-induced the production of intracellular ROS and the decrease of cell viability (**D**). Cells were pre-treated with NAC at a final concentration of 2.5 mM for 2 h, followed by co-treatment with CdSO_4_ at 10 μM for an additional 24 h. The ROS levels were quantified using a flow cytometry analysis (**C**). Cell viability was measured using the CCK-8 method (**D**). (**E**) malondialdehyde (MDA) levels. (**F**) the catalase (CAT) activities. (**G**) the superoxide dismutase (SOD) activities. All results are presented as mean ± SD (n = 3). ** *p* < 0.01 compared to that in the untreated control group; ^##^ *p* < 0.01 compared to the CdSO_4_ only-treated group.

Supplementary Figure S4A shows that the fluorescence intensities of DCFH-DA in HT22 cells treated with CdSO_4_ alone were significantly heightened. This effect was partially alleviated by co-treatment with EA in a dose-dependent manner. A similar finding was also detected in PC12 cells (Supplementary Figure S4B). Then, the quantitative analysis using flow cytometry revealed a notable increase in fluorescence intensities to 46% in the 10 μM CdSO_4_ treatment group in HT22 cells. Meanwhile, EA treatment at concentrations of 5, 10, and 20 μM led to significant reductions in fluorescence intensities to 30%, 26%, and 14% (all *p* < 0.01), respectively, compared to the group solely treated with CdSO_4_ (Figure 4A). Furthermore, the MitoSOX staining was performed. The results showed that the mitochondrial ROS levels were significantly increased to 2.6-fold, which were effectively decreased to 1.3- and 1.1-fold (both *p* < 0.01) by EA (at 20 μM) and Mito-TEMPO (at 5 μM) (Figure 4B). Antioxidant N-acetylcysteine (NAC) supplementation (at 2.5 mM) could effectively mitigate CdSO_4_-induced ROS production (Figure 4C) and partially alleviated CdSO_4_-induced cytotoxicity, resulting in an increase in the cell viability from 61% to 90% (*p* < 0.01; Figure 4D).

Additionally, the oxidative stress biomarkers, including MDA levels and the activities of SOD and CAT in HT22 cells, were evaluated. The results demonstrated that supplementation with EA effectively ameliorated Cd-induced oxidative damage. In Figure 4B–D, compared to the group exposed to 10 μM CdSO_4_ only, treatment with EA at concentrations of 5, 10 and 20 μM notably reduced MDA levels from 0.85 nmol/mg protein to 0.65, 0.60 and 0.39 nmol/mg protein (all *p* < 0.01), respectively. Moreover, EA treatment significantly increased CAT activities from 2.1 U/mg protein to 3.3, 4.5 and 5.1 U/mg protein (all *p* < 0.05 or 0.01), respectively, and elevated SOD activities from 24.2 U/mg protein increased to 35.2, 41.3 and 51.5 U/mg protein (all *p* < 0.05 or 0.01). Notably, EA treatment at the concentration of 20 μM did not alter the levels of MDA or the activities of CAT and SOD compared to the control group (Figure 4E–G).

### 3.4. EA Supplementation Attenuates CdSO_4_-Caused Mitochondrial Dysfunction

**Figure 5:**
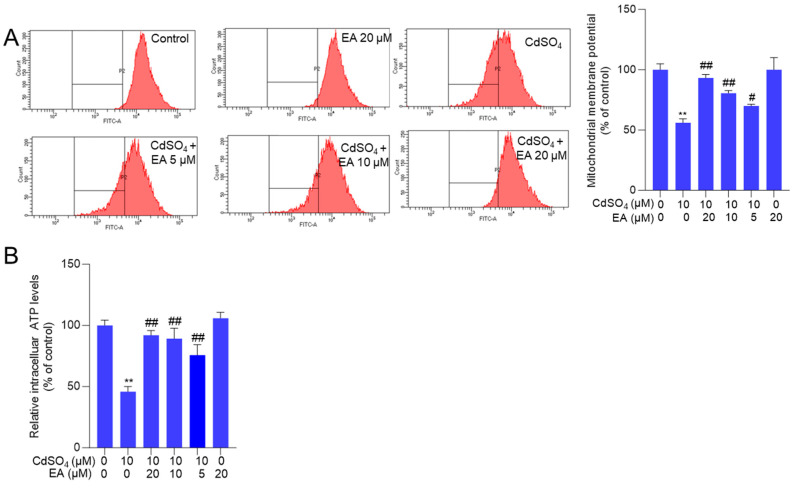
The changes in mitochondrial function. Cells were pre-treated with ellagic acid (EA) at 5, 10, or 20 μM for 2 h, followed by co-treatment with CdSO_4_ at 10 μM for an additional 24 h. (**A**) the changes in mitochondrial membrane potential were evaluated using Rh123 staining and flow cytometry analysis. The representative images (left) and quantitative analysis (right) were shown. (**B**) the intracellular ATP levels. All results are presented as mean ± SD (n = 3). ** *p* < 0.01, compared to that in the untreated control group; ^#^ *p* < 0.05, ^##^ *p* < 0.01 compared to the CdSO_4_ only-treated group.

Mitochondrial function was evaluated by measuring changes in mitochondrial membrane potential. In Figure 5A, EA treatment at concentrations of 5, 10, and 20 μM significantly increased the membrane potential from 56% to 70%, 81%, and 93% (all *p* < 0.05 or 0.01), respectively, compared to the group exposed to CdSO_4_ alone. Consistently, NAC supplementation significantly attenuated the loss of mitochondrial membrane potential induced by CdSO_4_ exposure (Supplementary Figure S5). Consistently, we also found that CdSO_4_ exposure significantly decreased the intracellular ATP levels to 46% (*p* < 0.01), and EA supplementation at 10 and 20 μM effectively restored the intracellular ATP levels to 89% and 92% (both *p* < 0.01) (Figure 5B). Additionally, EA treatment at a dose of 20 μM did not alter the mitochondrial membrane potential and the intracellular ATP levels compared to the untreated control group (Figure 5).

### 3.5. EA Supplementation Upregulates the Expression of Nrf2, HO-1, and Bcl-2 Proteins and Downregulates the Expression of Bax, p-JNK, p-p38, and p-ERK Proteins

**Figure 6:**
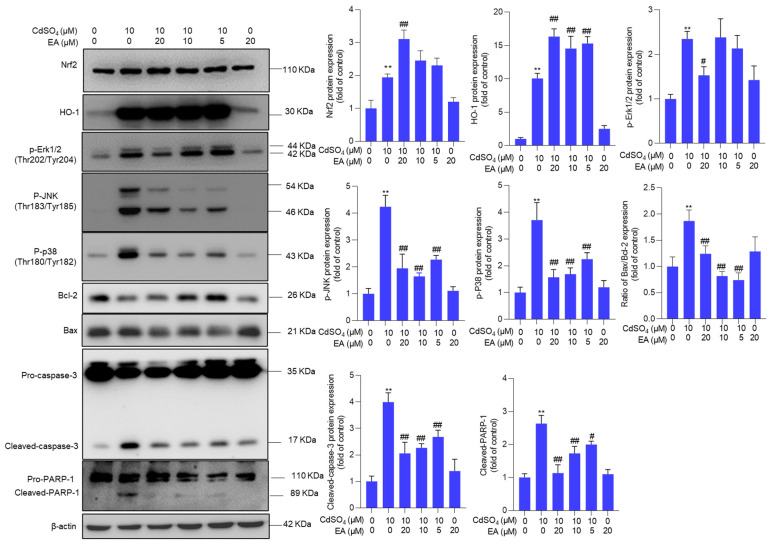
The expression of Nrf2, HO-1, p-Erk1/2, p-JNK, p-p38, Bax/Bcl-2 ratio, cleaved caspase-3, and cleaved PARP-1 proteins. Cells were pre-treated with ellagic acid (EA) at 5, 10, or 20 μM for 2 h, followed by co-treatment with CdSO_4_ at 10 μM for an additional 24 h. Protein expressions were assessed using the Western Blotting method. All results were presented as Mean ± SD (n = 3). Statistical significance levels were delineated as follows: ** *p* < 0.01 compared to the untreated control group; ^#^ *p* < 0.05 and ^##^ *p* < 0.01 compared to the CdSO_4_ only-treated group.

Compared to the untreated control group, exposure to 10 μM CdSO_4_ significantly upregulated the expression of Nrf2, HO-1, p-Erk1/2, p-JNK, p-p38, Bax/Bcl-2 ratio, cleaved caspase-3, and cleaved PARP-1 proteins by 1.9-, 10.1-, 2.4-, 4.3-, 3.7-, 1.9-, 4.0- and 2.6-fold, respectively (all *p* < 0.01) (Figure 6). These protein expressions were modulated by EA supplementation. EA supplementation at a dose of 20 μM plus CdSO_4_ treatment significantly increased the expression of Nrf2 and HO-1 proteins by 3.1- and 16.3-fold, respectively, while notably decreasing the expression of p-Erk1/2, p-JNK, p-p38, Bax/Bcl-2 ratio, cleaved caspase-3, and cleaved PARP-1 proteins to 1.5-, 2.0-, 1.6-, 1.2-, 2.1-, and 1.0-fold (all *p* < 0.01; Figure 6), respectively. Furthermore, we also found that EA supplementation could significantly upregulate the expression of Nrf2, HO-1, and Bcl-2 mRNAs and significantly downregulate the expression of Bax mRNA. As shown in Supplementary Figure S6, EA supplementation at 20 µM significantly upregulated the expression of Nrf2 mRNA from 1.9-fold to 3.6-fold, upregulated the expression of HO-1 from 4.6-fold to 10.1-fold, upregulated the expression of Bcl-2 from 0.4-fold to 0.9-fold, and significantly downregulated the expression of Bax from 2.3-fold to 1.2-fold, compared to those in the CdSO_4_-only group. EA alone treatment at 20 µM upregulated the expression of Nrf2 and HO-1 mRNAs but did not affect the expression of Bcl-2 and Bax mRNAs compared to those in the control group (Supplementary Figure S6).

We also confirmed the impact of oxidative stress on CdSO_4_-induced activation of the JNK and mitochondrial apoptotic pathways. As shown in Supplementary Figure S7, NAC supplementation led to a substantial decrease in the expression of p-JNK, the Bax/Bcl-2 ratio, cleaved caspase-3, and cleaved PARP-1 proteins. In the NAC and CdSO_4_ co-treatment group, the levels of p-JNK, the Bax/Bcl-2 ratio, cleaved caspase-3, and cleaved PARP-1 proteins nearly normalized, and they were decreased to 1.3-, 0.8-, 1.1-, and 1.1-fold (all *p* < 0.01) (Supplementary Figure S7), respectively. When compared to the control group, treatment with NAC alone did not affect the expression of the aforementioned proteins (Supplementary Figure S7).

### 3.6. The Pharmacological Inhibition of JNK Promotes CdSO_4_-Induced Cytotoxicity

**Figure 7:**
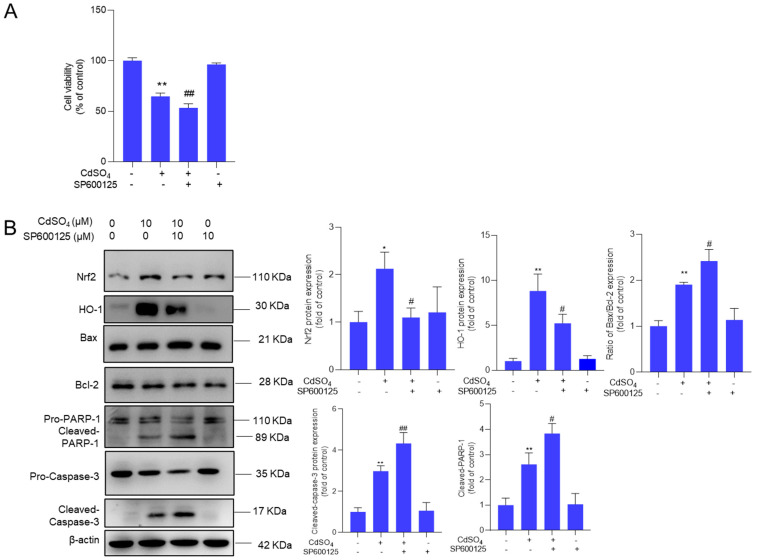
Pharmacological inhibition of JNK attenuates the activation of Nrf2/HO-1 and promotes CdSO_4_ exposure-induced cytotoxicity. HT22 cells were treated with the JNK inhibitor SP600125 at 10 μM for 2 h, then co-treated with CdSO_4_ at 10 μM for an additional 24 h. (**A**) the changes in cell viability were assessed. (**B**) the protein expressions were analyzed using the Western Blotting method. All results were presented as mean ± SD (n = 3). Significance levels were indicated as follows: * *p* < 0.05, ** *p* < 0.01 compared to the untreated control group; ^#^ *p* < 0.05, and ^##^ *p* < 0.01 compared to the CdSO_4_ only-treated group.

We further investigated the impact of the MAPK pathway on CdSO_4_ exposure-induced cytotoxicity. We found that pharmacological inhibitions of p38 and ERK pathways by SB203580 and ASN007 did not affect CdSO_4_-induced cytotoxicity in HT22 cells (Supplementary Figure S8). However, the pharmacological inhibition of JNK by SP600125 could significantly exacerbate CdSO_4_ exposure-induced cytotoxicity (Figure 7A). Additionally, JNK inhibition led to a substantial decrease in the expression of Nrf2 and HO-1 proteins, which were decreased to 1.1- and 5.2-fold (both *p* < 0.05), respectively. On the contrary, compared to the group exposed to CdSO_4_ only, JNK inhibition significantly increased the expression of the Bax/Bcl-2 ratio, cleaved caspase-3, and cleaved PARP-1 proteins to 2.4-fold (*p* < 0.05), 4.3- fold (*p* < 0.01), and 3.8-fold (*p* < 0.05) (Figure 7B).

## 4. Discussion

Neurotoxicity is one of the multiple toxic effects caused by CdSO_4_ exposure [18]. Previous studies demonstrated that CdSO_4_ exposure at the range of 0.625–20 μM could induce marked decreases in the cell viability in PC12 cells and HT22 cells [13,27]. Consistent with a previous study [13], our current study reveals that exposure to CdSO_4_ at concentrations ranging from 0.625 to 40 μM resulted in dose-dependent cell death in HT22 cells. Previous studies have reported that the different test methods have different sensitivity to Cd-induced cytotoxicity [28]. One non-negligible reason is that some heavy metals can affect the sensitivity of CCK-8 detection, and it might be increased with the increase of heavy metal concentration. In the present study, we used two methods, i.e., the CCK-8 and LDH methods, to assess the cytotoxicity and the protective effects of EA. Our results found that the LDH method is more sensitive than the CCK-8 method in Cd-treated HT22 cells (Figure 2). Meanwhile, we found that both two methods demonstrated the protective effects of EA on Cd-induced cytotoxicity (Figure 2). Similarly, EA supplementation at 5–20 μM could provide protection against Cd-induced cytotoxicity in PC12 cells (Supplementary Figure S1). Furthermore, we observed a dose-dependent inhibition by EA supplementation on CdSO_4_ exposure-induced oxidative stress, cell apoptosis, and mitochondrial dysfunction. These effects may be linked to EA’s ability to scavenge free radicals, enhance antioxidant defense function, inhibit the JNK pathway, and activate the Nrf2/HO-1 pathway (Figure 3, Figure 4, Figure 5, Figure 6, Figure 7 and Supplementary Figures S2–S8).

Apoptosis, a form of programmed cell death, can be induced by various drugs or environmental toxins, such as copper, cisplatin, and T-2 toxin [26,29,30,31]. In this study, we observed that exposure to CdSO_4_ could initiate cell apoptosis (Figure 3), consistent with prior research [32,33]. Furthermore, our findings revealed that EA supplementation effectively ameliorated CdSO_4_-induced cell apoptosis in HT22 and PC12 cells (Figure 3 and Supplementary Figures S2 and S3). Several previous studies demonstrated that EA supplementation can reduce drugs or toxins-induced apoptotic cell death in vitro and in vivo [34,35,36]. These findings suggest that EA’s protective effects against CdSO_4_-induced cytotoxicity involve the inhibition of apoptosis.

Apoptotic cell death could be triggered by multiple signals, including ROS, p53, and other apoptosis-related factors [26,37]. A prior study demonstrated that exposure to CdSO_4_ can induce excessive ROS production, leading to direct damage to intracellular macromolecules (such as DNA, lipids, and proteins) and subcellular organelles (such as mitochondria, endoplasmic reticulum, and lysosomes), ultimately culminating in cell apoptosis [38]. Our current findings reveal that CdSO_4_ exposure significantly elevates intracellular ROS in HT22 cells and PC12 cells (Figure 4 and Supplementary Figure S4). Meanwhile, CdSO_4_ exposure significantly upregulated the MDA levels while simultaneously decreasing the activities of SOD and CAT in HT22 cells (Figure 4). We also found that CdSO_4_ exposure could significantly upregulate the mitochondrial ROS levels (Figure 4). Similarly, Hyun et al. showed that Cd exposure at 10–40 μM for 24 h could significantly upregulate the mitochondrial ROS levels in human prostate stromal cells and mouse embryonic fibroblasts [39]. These data suggested that CdSO_4_ exposure-induced excessive production is partly dependent on the production of mitochondrial ROS. SOD and CAT, as antioxidant enzymes, play crucial roles in combating oxidative stress by scavenging intracellular superoxide and hydroxyl radicals [26]. Increased MDA levels serve as an essential indicator of membrane lipid peroxidation [25]. These results indicate that CdSO_4_ exposure elicits oxidative stress damage in HT22 cells. Notably, EA supplementation effectively attenuates CdSO_4_-induced ROS and mitochondrial ROS productions and enhances SOD and CAT activities (Figure 4). Previous studies by Firdaus et al. demonstrated that EA pre-treatment at 10–20 μM can inhibit ROS production and apoptosis induced by arsenic trioxide in human neuroblastoma SH-SY5Y cells [40]. Similarly, Ding et al. reported that EA treatment at 15–30 μM reduces ROS production and MDA levels caused by high glucose exposure while improving SOD activities in HepG2 cells [41]. Moreover, Zhao et al. showed that EA supplementation at doses of 50–100 mg/kg/day for four weeks upregulates antioxidant enzymes, including CAT, SOD, and GSH-PX, to ameliorate ethanol exposure-induced liver injury in a mouse model [42]. Collectively, these findings suggest that EA supplementation effectively reduces CdSO_4_-induced apoptosis by alleviating oxidative stress and mitochondrial ROS production. Furthermore, our study found that ATP production was significantly decreased after CdSO_4_ exposure, indicating mitochondrial dysfunction (Figure 5). It is known that mitochondrial respiration mainly relies on the enzymatic activities of five mitochondrial complexes that couple electron transport with proton pumping, finally leading to ATP synthesis [43]. Several previous studies also reported that Cd is a potent uncoupling agent and can inhibit mitochondrial respiratory chain activities by interacting with CI at the Q site and NADH site and complexes I (CI), CII, and CIII at the Fe–S cluster [14,44,45,46]. Our results also showed that EA treatment for 24 h could effectively restore CdSO_4_ exposure-induced loss of intracellular ATP (Figure 5). These data suggested that EA supplementation may provide protection for CdSO_4_ exposure-induced mitochondrial dysfunction via the inhibition of oxidative stress. The inhibition of complexes I and II could induce ROS generation [47,48]. Khodaei et al.’s findings showed that EA supplementation could inhibit cuprizone exposure-induced loss of CII, CIII, and CIV proteins in mouse muscle tissue [49]. Therefore, the regulation of EA on mitochondrial complexes may participate in its protective effects against CdSO_4_-induced mitochondrial dysfunction. This is a limitation of our current study. Further investigation is warranted to elucidate the underlying molecular mechanisms.

Mitochondria serve not only as producers but also as targets of ROS [50]. Bax, a pro-apoptotic protein, contrasts with Bcl-2, an anti-apoptotic protein [50]. The mitochondrial membrane potential assessment stands as a pivotal indicator of mitochondrial dysfunction [26]. Deng et al. observed a significant loss of mitochondrial membrane potential in N2a neuronal cells upon exposure to cadmium chloride (CdCl_2_) [1]. The elevation in the Bax/Bcl-2 ratio can instigate mitochondrial dysfunction, triggering the release of CytC and subsequently activating caspases-9 and -3 in a cascading fashion, culminating in cell apoptosis [51]. Poly (ADP-ribose) polymerase-1 (PARP-1) acts as a target of activated caspases-3 in caspase-dependent apoptosis, with cleaved PARP-1 suppressing DNA repair and serving as a key marker of apoptosis [52]. Our study demonstrates that CdSO_4_ exposure notably diminishes mitochondrial membrane potential, leading to an increase in the Bax/Bcl-2 ratio, ultimately resulting in the upregulation of cleaved caspase-3 and cleaved PARP-1 protein expression (Figure 5 and Figure 6 and Supplementary Figure S6). Pan-caspase inhibitors could markedly revise CdSO_4_-induced cell apoptosis (Supplementary Figure S2), indicating that CdSO_4_-induced apoptotic cell death is caspase-dependent. EA supplementation partially reduces these CdSO_4_-induced alterations (Figure 5 and Figure 6 and Supplementary Figure S6). Furthermore, our data reveals that the antioxidant NAC supplementation significantly attenuates CdSO_4_-induced cytotoxicity by suppressing ROS production and the mitochondrial apoptotic pathway (Figure 4 and Supplementary Figures S5 and S7). Therefore, these findings suggest that EA supplementation could effectively safeguard against CdSO_4_-induced apoptosis by hindering mitochondrial dysfunction and the mitochondrial apoptotic pathway in HT22 cells, potentially attributable to its role in oxidative stress modulation.

Nrf2, a crucial transcription factor often referred to as a “housekeeper,” responds to oxidative stress and inflammatory damage [53,54]. Studies have highlighted that Nrf2 activation exerts a protective effect against Cd-induced cytotoxicity and tissue damage [55,56]. Consistent with prior findings, our study demonstrated a significant upregulation of Nrf2 mRNA and protein expression and its downstream gene HO-1 following CdSO_4_ exposure, with further enhancements observed upon co-treatment with EA (Figure 6 and Supplementary Figure S6). Recent research has underscored Nrf2 as a vital target of EA, showing that EA promotes nuclear Nrf2 expression by inhibiting Keap1 expression [57]. Notably, Nrf2 knockout partially attenuated the protective effects of EA against rotenone-induced neurotoxicity in a mouse model [22]. Our current data strongly suggest that Nrf2 activation contributes to the EA-mediated protection against CdSO_4_-induced apoptosis.

Previous studies have shown that Cd exposure can promptly activate the MAPK pathway, leading to a significant upregulation of p-Erk1/2, p-JNK, and p-p38 proteins in various cell types, including PC12 and SH-SY5Y neuronal cells and human bronchial epithelial cells (BEAS-2B) [58,59,60]. In these cell models, researchers observed that the inhibition of p-Erk1/2 and p-JNK partially attenuated Cd exposure-induced cell apoptosis [58,59,60]. Conversely, Liu et al. reported that inhibiting JNK did not influence Cd exposure-induced cell death in BmE cells, a silkworm embryonic cell line [61]. Given its diverse functions, the MAPK pathway typically exhibits a dual role in regulating cell apoptosis [62]. For instance, Hu et al. demonstrated that JNK-mediated activation of the Nrf2 pathway contributed to the protective effects of coptisine against 2,2′-azodiisobutyramidine dihydrochloride-induced oxidative stress damage in zebrafish embryos [62]. In the present study, we observed that JNK inhibition, not p38 and ERK inhibition, could promote CdSO_4_-induced cytotoxicity at 24 h (Supplementary Figure S8). Moreover, JNK inhibition by SP600125 markedly suppresses the expression of Nrf2 and HO-1 proteins, consequently exacerbating the mitochondrial apoptotic pathway and ultimately exacerbating CdSO_4_ exposure-induced cell death (Figure 7). We also found that blocking oxidative stress with NAC substantially inhibited JNK activation (Supplementary Figure S7), suggesting that oxidative stress is a crucial upstream regulator of the JNK pathway in CdSO_4_-induced cytotoxicity. For the MAPK pathway, the time profile of activation is usually responsible for the biological outcome. In the current study, we just tested the effects of the MAPK pathway on CdSO_4_-induced cytotoxicity at 24 h. The dynamic changes of the MAPK pathway in response to CdSO_4_ and how it mediates the protective effects of EA are still unknown. This is another limitation of the present study.

Importantly, EA has a higher safety for humans and animals and has been used in food production as a food additive [63,64]. Tasaki et al. found that the no-observed-effect levels (via the 90-day sub-chronic toxicity study) of EA in male or female rats are 3011 mg/kg body weight/day and 3254 mg/kg body weight/day, respectively [63]. Various studies indicate that oral EA supplementation at 10–100 mg/kg body weight per day could provide neuroprotection for drugs or environmental toxins (such as rotenone, 6-hydroxydopamine, and 1-methyl-4-phenyl 1,2,3,6 tetrahydropyridine)-induced neurotoxicity in rats or mice [22,36,65,66]. A recent human trial showed that EA intervention (90 mg twice a day for 12 weeks) could provide a favorable effect on depression in multiple sclerosis [67]. The information indicated that oral supplementation of EA is safer. Taken together, these findings suggest that EA may be a potential protective agent against Cd-induced neurotoxicity, and further commercial development could be considered.

## 5. Conclusions

**Figure 8:**
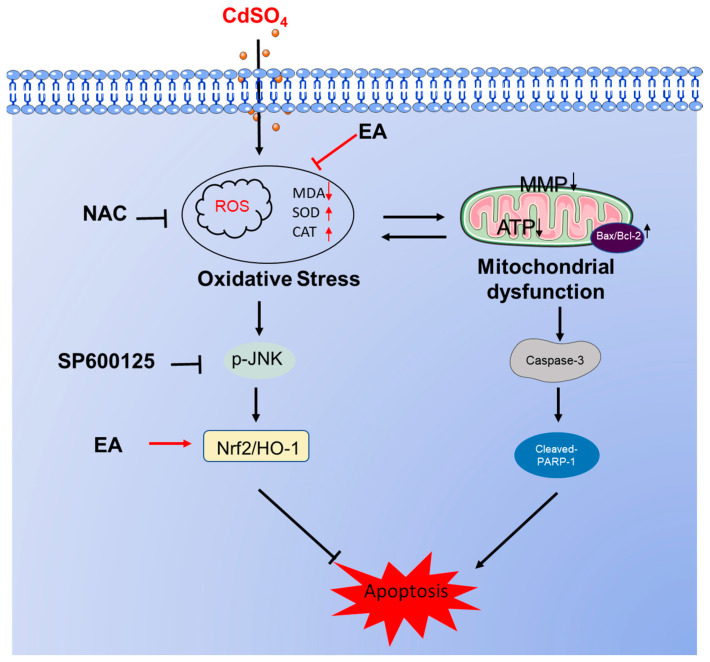
A potential proposed model of EA supplementation on CdSO_4_ exposure-caused apoptosis. CdSO_4_ exposure leads to the generation of reactive oxygen species (ROS) and lipid peroxidation while reducing the activities of antioxidant enzymes SOD and CAT, resulting in oxidative stress damage. Elevated ROS levels may disrupt mitochondrial function, triggering the activation of the mitochondrial apoptotic pathway and eventually culminating in apoptosis. Furthermore, activated JNK exerts a protective effect by stimulating the Nrf2/HO-1 pathway.

In summary, our results demonstrate that EA supplementation effectively reduced CdSO_4_-induced cytotoxicity in HT22 cells by inhibiting oxidative stress and apoptosis, potentially via the activation of the Nrf2/HO-1 pathway. Additionally, the activation of the JNK pathway in response to CdSO_4_ exposure also confers protection via the Nrf2/HO-1 pathway. A proposed model illustrating the impact of EA supplementation on CdSO_4_-induced apoptosis is presented in Figure 8. This study highlights EA as a promising candidate for combating Cd-induced cytotoxicity and neurotoxicity.

## Data Availability

The data are contained within the article and Supplementary Materials.

## Supplementary Materials

The following supporting information can be downloaded at: https://www.mdpi.com/article/10.3390/antiox13111296/s1, Supplementary Figure S1 The morphology changes (left) and the results of cell viabilities (right) in PC12 cells after CdSO4 treatment with or without ellagic acid (EA). Supplementary Figure S2 Pan-caspase inhibitor Z-VAD-FMK suppresses CdSO4-induced apoptosis in HT22 cells. Supplementary Figure S3 Ellagic acid (EA) supplementation attenuates CdSO4-induced cell apoptosis in PC12 cells. Supplementary Figure S4 Representative images of DCFH-DA staining in HT22 cells (A) and PC12 cells (B). Supplementary Figure S5 NAC supplementation attenuates CdSO4-induced alterations in mitochondrial membrane potential. Supplementary Figure S6 The mRNA expression of Nrf2 (A), HO-1 (B), Bcl-2 (C), and Bax (D) genes. Supplementary Figure S7 NAC supplementation attenuates CdSO4-induced activation of JNK and mitochondrial apoptotic pathways. Supplementary Figure S8 Effects of pharmacological inhibition of p38 (A) and Erk (B) pathways by SB203580 and ASN007 on CdSO4 exposure-induced cytotoxicity. References [24,26] are cited in the supplementary materials.
