# Supplementary material for: Ellagic Acid Reduces Cadmium Exposure-Induced Apoptosis in HT22 Cells via Inhibiting Oxidative Stress and Mitochondrial Dysfunction and Activating Nrf2/HO-1 Pathway
Source: Antioxidants (Basel). 2024 Oct 25;13(11):1296. doi: 10.3390/antiox13111296 (PMC11590970; doi:10.3390/antiox13111296)
Supplement: Supplementary file 1 [file antioxidants-13-01296-s001.zip › antioxidants-3228297-supplementary.pdf]

Ellagic acid reduces cadmium exposure-induced apoptosis in HT22 cells via inhibiting oxidative stress and mitochondrial dysfunction and activating Nrf2/HO-1 pathway

Yue Liu[1,2], Chunhong Chen[1,2], Zhihui Hao[1,2], Jianzhong Shen[1,2], Shusheng Tang[1,2*], Chongshan Dai[1,2*]

[1] National Key Laboratory of Veterinary Public Health and Safety, College of Veterinary Medicine, China Agricultural University, Beijing, 100193, P.R. China.

[2] Key Biology Laboratory of Chinese Veterinary Medicine, Ministry of Agriculture and Rural Affairs, Beijing, 100193, P.R. China.

*Correspondences: Chongshan Dai, E-mail: daichongshan@cau.edu.cn; Shusheng Tang, E-mail: tssfj@cau.edu.cn.

Supplemental Materials and Methods

2.4 LDH measurement

The toxic effects of cadmium sulfate ($CdSO_4$) exposure on cell membranes of HT22 cells were measured using the lactate dehydrogenase (LDH) release method. The detailed protocol follows the instructions provided by a commercially available LDH kit (Beyotime Biotechnology Company, Haimen, China). In brief, a 96-well cell culture plate was used and 1×10^4 cells were seeded per well. After 24 hours of cultivation, cells were treated with $CdSO_4$ at various concentrations of 0.625, 1.25, 2.5, 5, and 10 μM for 24 hours. After treatment, the culture medium was collected and centrifuged at 400 rpm for 5 min. Then, the supernatants were collected and the levels of LDH were determined. The final values were normalized to the control group.

To investigate the effects of ellagic acid (EA) supplementation on $CdSO_4$-induced cell membrane damage, cells were pretreated with EA at the final concentrations of 5, 10, and 20 μM for 2 hours, then were co-treated with or without $CdSO_4$ at 10 μM for another 24 hours. After treatment, the LDH levels were measured.

2.6 Measurement of intracellular ROS levels and oxidative stress biomarkers

The levels of intracellular ROS were measured using ROS-specific fluorescent dye 2,7-dichlorofluorescein diacetate (DCFH-DA) (Beyotime, Haimen, China), according to the description from a previous study (Dai et al., 2018). Briefly, 1×10^5 cells per well were seeded into a 12-well plate. After 24 h, cells were pretreated with EA at 5, 10, and 20 μM for 2 hours, then were co-treated with or without $CdSO_4$ at 10 μM. After 24 hours, cells were washed twice with PBS (5 minutes per time) and incubated with 10

μM DCFH-DA for 30 min at 37°C in the dark. Finally, cells were washed twice with PBS (5 minutes per time). ROS levels were visualized under a fluorescence microscope (Leica, Wetzlar, Germany) with excitation and emission wavelengths set at 490 nm and 525 nm. The fluorescence intensity was quantified using flow cytometry (Becton Dickinson, San Jose, CA, USA).

Meanwhile, the biomarkers of intracellular oxidative stress, including malondialdehyde (MDA) levels, and catalase (CAT) and superoxide dismutase (SOD) activities were measured using the commercially available MDA, CAT, and SOD Kits (Nanjing Jiancheng Biological Engineering, Nanjing, China), respectively. The detailed protocol was strictly according to the manufacturer's instructions, respectively.

2.7 Measurement of intracellular ATP levels

The intracellular ATP levels in HT22 cells were measured with the Enhanced ATP Assay Kit (Beyotime, Haimen, China), according to the commercial kit instructions. Briefly, 2 × 105 cells/well were plated into a 12-well plate, then cells were pretreated with EA at the doses of 5, 10, and 20 μM for 2 hours, then co-treated with $CdSO_4$ at dose of 10 μM for additional 24 hours. After treatment, cells were lysed using the 100 μL lysis buffer provided by the kit and the lysates were centrifugated at 14,000× g for 10 min at 4 °C. Then, the supernatants were collected and mixed with ATP detection working liquid for measurement. The values were read using a microplate luminometer (Tecan Trading AG, Männedorf, Switzerland).

2.8 Western Blotting

The expression of proteins was analyzed using Western Blotting, according to the

detailed protocol provided in a previous study (Li et al., 2024). The cell samples were homogenized in ice-cold lysis buffer (it includes 100 mM Tris-HCl, 2% [mass/volume] SDS, 10% [vol/vol] glycerol and pH 7.4; 1 mM PMSF, 1 μg/mL aprotinin, 1 μg/mL leupeptin, and 1 μg/mL pepstatin A) and then ultrasonicated (i.e., 5 s ultrasonication and 6 s pause in each cycle for 5 times, power 30 W) using an Ultrasonic Processor (Branson, MO, USA). Then, the samples were divided into two parts. Cell samples were centrifuged at 145,000 × *g* for 15 min at 4°C, and protein concentration was measured using the BCA protein assay kit. For the measurement of mitochondrial complexes, the samples were added 4 × loading buffer (the final concentration of work solution is 1 ×) and incubated at 45 °C for 10 min. For the other protein examination, samples were incubated at 95 °C for 10 min. Western blotting was conducted with 20 μg protein per lane. The primary rabbit polyclonal antibodies against phosphorylated (p)-p38 (Thr180/Tyr182), p-JNK (Thr183/Tyr185), p-ERK1/2 (Thr202/Tyr204) (1:1000 dilution; CST company, Beverly, MA, USA), Nrf2, HO-1, Bax, Bcl-2, caspase-3 (1:1000 dilution; Proteintech, Chicago, IL, USA), and mouse monoclonal antibody against β-actin (1:1000 dilution; Santa Cruz, CA, USA) were used. The anti-Total OXPHOS Cocktail antibody was purchased from Abcam (Shanghai, China). Secondary antibodies, including goat anti-rabbit IgG (1:5,000) or rabbit anti-mouse IgG (1:5,000) were incubated for 1 hour at room temperature. The protein expression results were normalized to β-actin and analyzed using ImageJ (National Institute of Mental Health, Bethesda, MD, USA).

2.9 qRT-PCR analysis

Gene expression analysis was performed by qRT-PCR. In brief, total RNA was isolated using a commercial Fast Pure® Cell/Tissue Total RNA Isolation Kit V2 (Vazyme Biotech Co.,Ltd). The quality of isolated RNA was evaluated by the optical density at 260/280 nm (all values are between 1.9~2.1). Approximately 500 ng of total RNA was subjected to reverse transcription to produce cDNA by using the Prime Script RT-PCR kit (Takara, Dalian, China). The detail primer information was as followed: Nrf2, Forward: 5′-CAC ATT CCC AAA CAA GAT GC-3′, Reverse: 5′-TCT TTT TCC AGC GAG GAG AT-3′; HO-1, Forward: 5′-CGT GCT CGA ATG AAC ACT CT-3; Reverse: 5′-GGA AGC TGA GAG TGA GGA CC-3′; Bax, Forward: 5′-AAACTGGTGCTCAAGGCCCT-3′, Reverse: 5′-AGCAGCCGCTCACGGAG-3′; β-actin, Forward: 5′-GCC CTG AGG CTC TTT TCC A-3′, Reverse: 5′-GTT GGC ATA GAG GTC TTT ACG GAT-3′. β-actin was used as an internal control, and $2^{-\Delta\Delta Ct}$ method was used to calculate the fold change of gene expression.

References

Dai, C., Ciccotosto, G.D., Cappai, R., Tang, S., Li, D., Xie, S., Xiao, X., Velkov, T., 2018. Curcumin Attenuates Colistin-Induced Neurotoxicity in N2a Cells via Anti-inflammatory Activity, Suppression of Oxidative Stress, and Apoptosis. Mol Neurobiol 55, 421-434.

Li, M., Tang, S., Velkov, T., Shen, J., Dai, C., 2024. Copper exposure induces mitochondrial dysfunction and hepatotoxicity via the induction of oxidative stress and PERK/ATF4 -mediated endoplasmic reticulum stress. Environ Pollut 352, 124145.

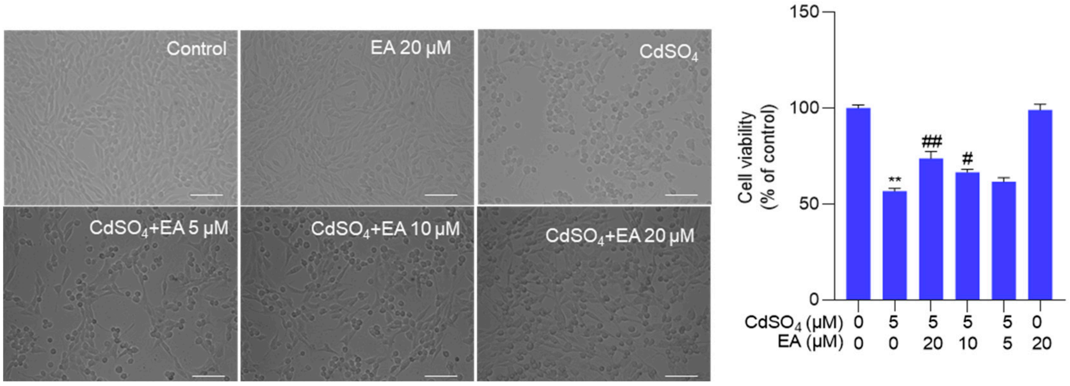

Suppl. Figure S1 The morphology changes (left) and the results of cell viabilities (right) in PC12 cells after $CdSO_4$ treatment with or without ellagic acid (EA). Cells were pre-treated with EA at 5, 10, or 20 μM for 2 hours, then cells were co-treated with $CdSO_4$ at 5 μM for additional 24 hours. After treatment, the cell viabilities were examined using the CCK-8 method. Compared to the untreated control group, $^{**}P < 0.01$; compared to the CdSO4 only-treated group, $^{\#}P < 0.05$ and $^{\#\#}P < 0.01$. Bar = 50 μm.

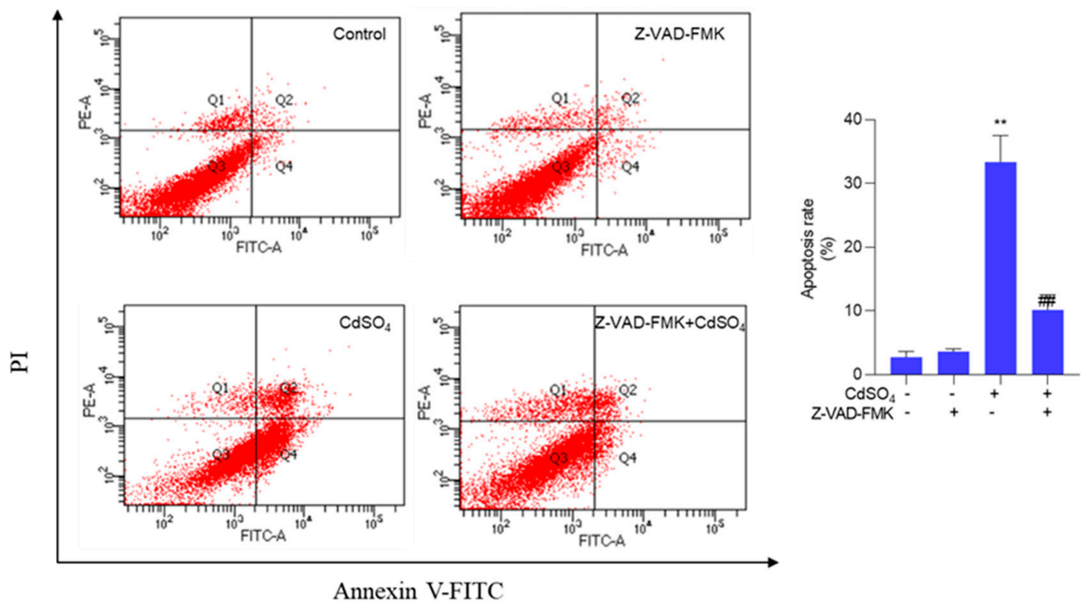

Suppl. Figure S2 Pan-caspase inhibitor Z-VAD-FMK suppresses $CdSO_4$-induced apoptosis in HT22 cells**.** Cells were pre-treated with Z-VAD-FMK at a final concentration of 20 μM for 2 hours, followed by co-treatment with $CdSO_4$ at 10 μM for additional 24 hours. The apoptosis rates were measured through Annexin V-FITC staining combined with flow cytometry analysis. Representative images of the flow cytometry analysis (left) and quantitative results (right) were displayed. All data are presented as mean ± SD (n = 3). $^{**}P < 0.01$ compared to the untreated control group; $^{\#\#}P < 0.01$ compared to the $CdSO_4$ only-treated group.

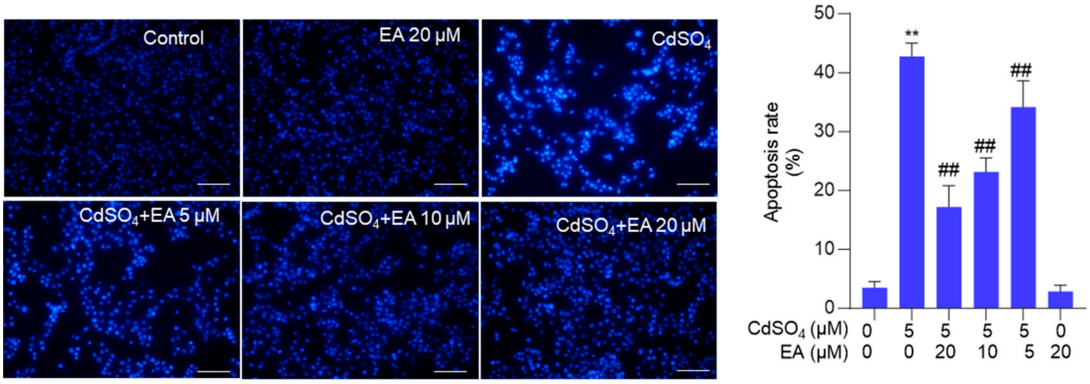

Suppl. Figure S3 Ellagic acid (EA) supplementation attenuates $CdSO_4$-induced cell apoptosis in PC12 cells. Cells were pre-treated with EA at final concentrations of 5, 10, or 20 μM for 2 hours, then cells were co-treated with $CdSO_4$ at 5 μM for 24 hours. The apoptotic rates were measured using the Hoechst 33342 staining method. Representative images (left) and quantitative results (right) are displayed. $^{**}P < 0.01$ compared to that in the untreated control group; $^{\#\#}P < 0.01$ compared to the $CdSO_4$ only-treated group. Bar = 50 μm.

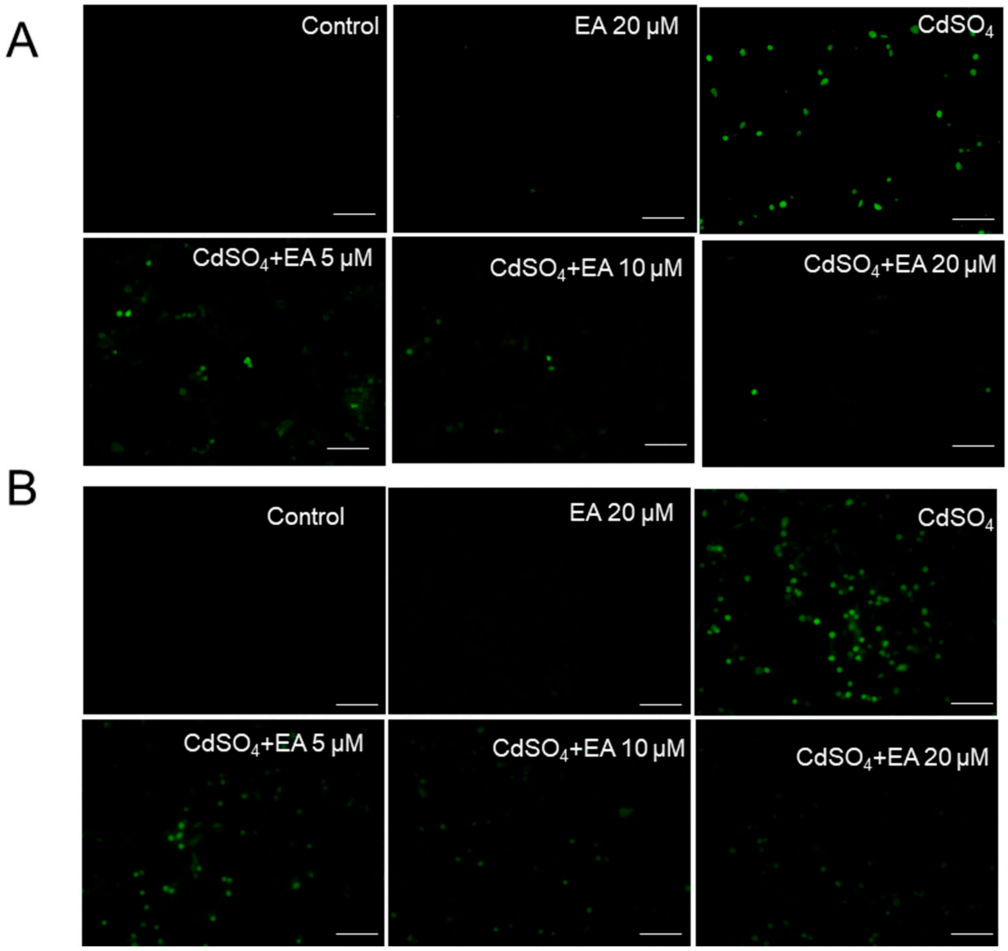

Suppl. Figure S4 Representative images of DCFH-DA staining in HT22 cells (A) and PC12 cells (B). Cells were pre-treated with ellagic acid (EA) at 5, 10, or 20 μM for 2 hours, followed by co-treatment with $CdSO_4$ at 5 or 10 μM for 24 hours in HT22 or PC12 cells. The ROS levels were visualized under a fluorescence microscope (Leica, Wetzlar, Germany) with excitation and emission wavelengths set at 490 nm and 525 nm. Bar = 50 μm.

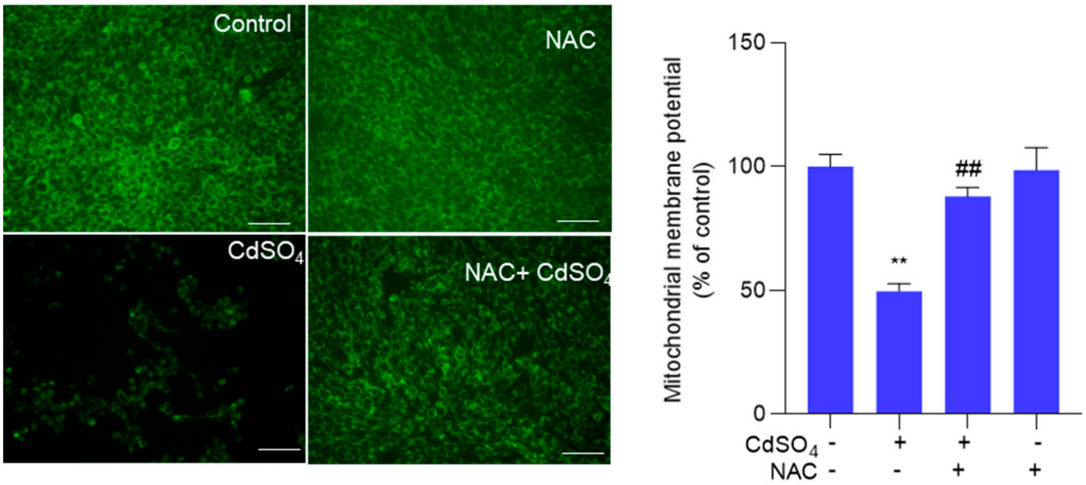

Suppl. Figure S5 NAC supplementation attenuates $CdSO_4$ -induced alterations in mitochondrial membrane potential. The representative images (left) and quantitative analysis (right) were shown. Data were presented as mean ± SD (n = 3). **$P < 0.01$, compared to the untreated control group; #$P < 0.05$, and ##$P < 0.01$, compared to the CdSO4 only-treated group. Bar = 50 μm.

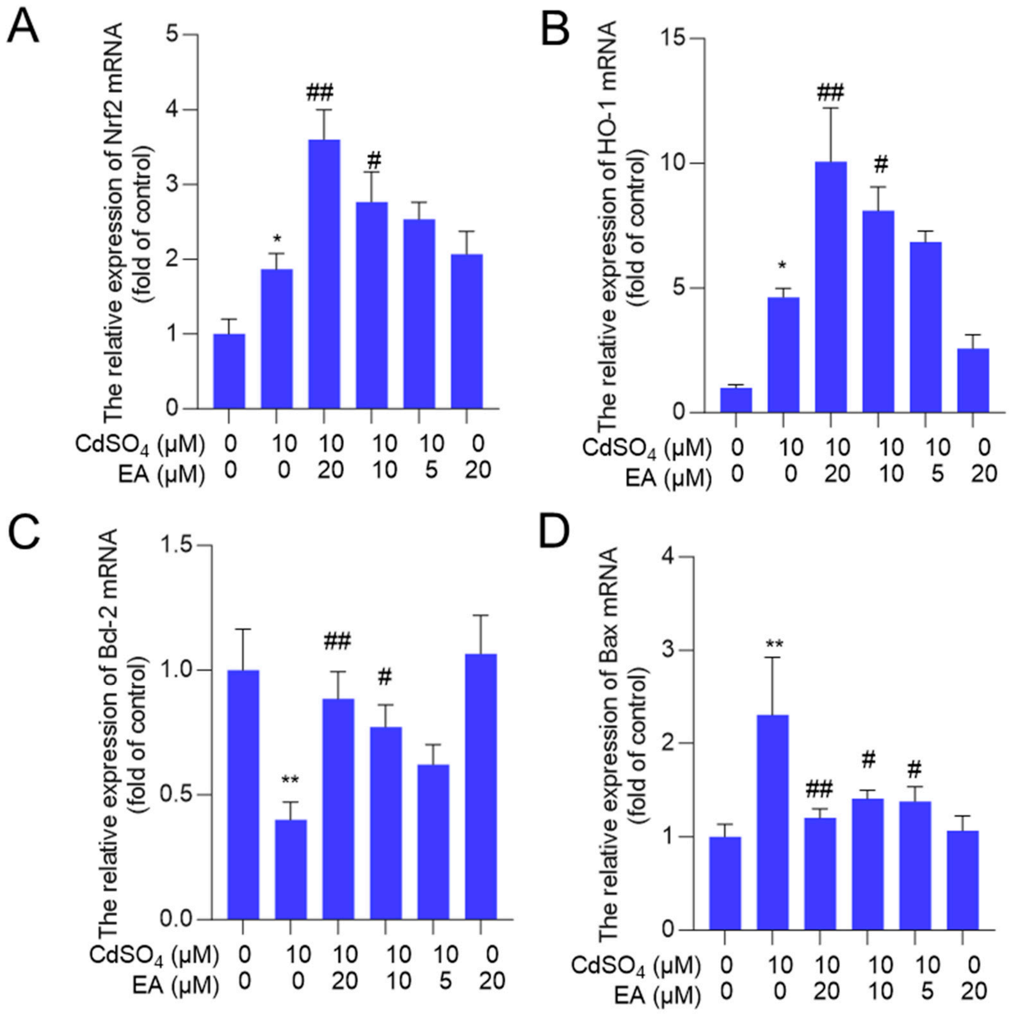

Suppl. Figure S6 The mRNA expression of Nrf2 (A), HO-1 (B), Bcl-2 (C), and Bax (D) genes. Cells were pre-treated with ellagic acid (EA) at 5, 10, or 20 μM for 2 hours, followed by co-treatment with CdSO4 at 10 μM for an additional 24 hours. The mRNA expression was measured using RT-PCR. All results were presented as Mean ± SD (n = 3). Statistical significance levels were delineated as follows: **P < 0.01 compared to the untreated control group; #P < 0.05 and ##P < 0.01 compared to the CdSO4 only-treated group.

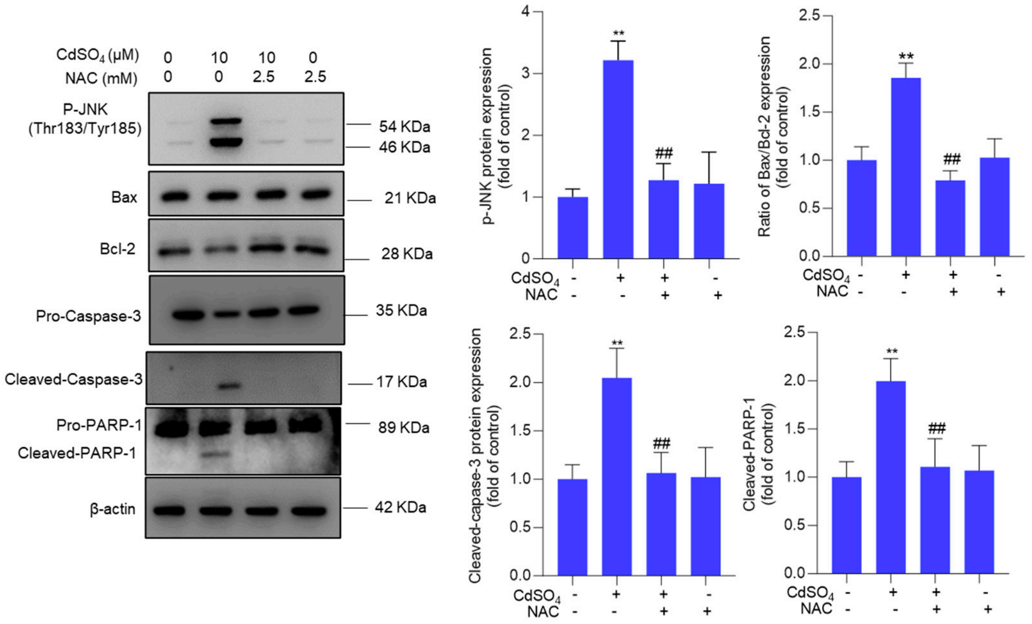

Suppl. Figure S7 NAC supplementation attenuates CdSO4-induced the activation of JNK and mitochondrial apoptotic pathways. The protein expressions were assessed using the Western Blotting method. Data were presented as mean ± SD (n = 3). $^{**}P < 0.01$, compared to the untreated control group; $\#P < 0.05$, and $\#\#P < 0.01$, compared to the CdSO4 only-treated group.

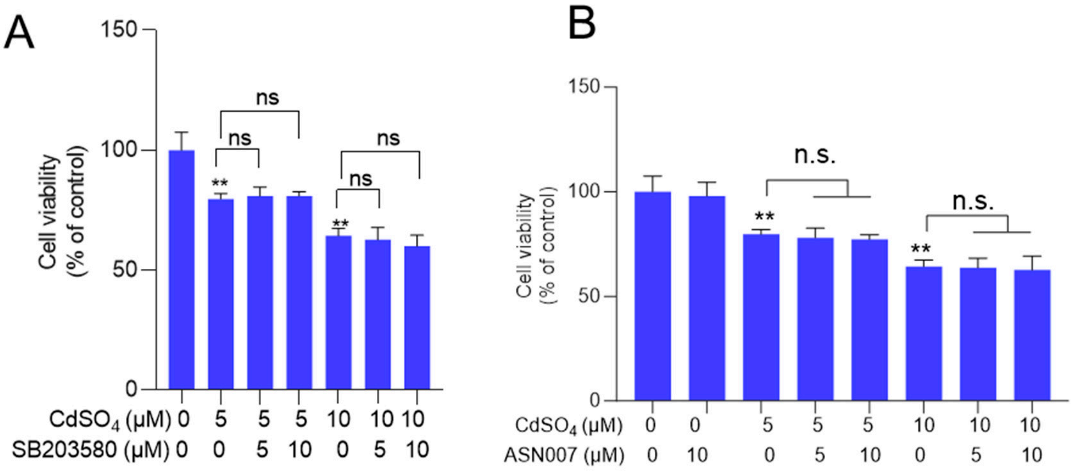

Suppl. Figure S8 Effects of pharmacological inhibition of p38 (A) and Erk (B) pathways by SB203580 and ASN007 on $CdSO_4$ exposure-induced cytotoxicity. All data are presented as mean ± SD (n = 5). ***P* < 0.01, compared to the untreated control group. n.s., no significance.
